# Epistemic values and the Big Five: Personality characteristics of those who ascribe personal and moral value to epistemic rationality

**DOI:** 10.1371/journal.pone.0258228

**Published:** 2021-10-05

**Authors:** Tomas Ståhl, James Turner

**Affiliations:** Department of Psychology, University of Illinois at Chicago, Chicago, Illinois, United States of America; Aalborg University, DENMARK

## Abstract

People differ in how much personal importance, and moral relevance, they ascribe to epistemic rationality. These stable individual differences can be assessed using the Importance of Rationality Scale (IRS), and Moralized Rationality Scale (MRS). Furthermore, these individual differences are conceptually distinct, and associated with different cognitive, affective, and behavioral outcomes. However, little is known about what signifies and differentiates people who score high (vs. low) on the IRS and MRS respectively, and where these individual differences stem from. In the present research we begin to address these questions by examining how these epistemic values relate to the Big Five personality traits. Two studies consistently show that both the IRS and MRS are positively related to Openness to experience. However, only the MRS is negatively associated with Agreeableness, and only the IRS is positively associated with Conscientiousness.

## Introduction

People differ in the extent to which they ascribe value to open-minded thinking about evidence, and epistemic rationality [1–4]. Epistemic rationality concerns the extent to which one’s beliefs accurately map onto the actual structure of the world, or our best available knowledge about the structure of reality [5]. Notably, people do not only differ in how important it is for them personally to be epistemically rational, but also in the extent to which they view epistemic rationality as a moral issue. The personal and moral value people ascribe to epistemic rationality are related—but conceptually distinct—stable individual differences that can be reliably assessed with the Importance of Rationality Scale (IRS) and Moralized Rationality Scale (MRS), respectively [4]. The distinction is not trivial, as each of these individual difference variables is associated with distinct processes and outcomes. The IRS has been uniquely linked to analytic thinking [6,7], and more strongly linked than the MRS to belief in science [4]. However, the MRS is uniquely associated with harsh moral judgments, and behavioral intentions, such as to socially distance oneself from people who rely on epistemically irrational (vs. rational) beliefs, as well as a willingness to support organizations that work to prevent the spread of irrational beliefs [4]. But where do these individual differences stem from, and what are the psychological profiles of people who view epistemic rationality as an important moral (vs. personal) value? In the present research we begin to address these questions, as we examine how these personal and moral epistemic values relate to the Big Five personality traits.

## Thinking dispositions and epistemic values

Psychologists have long studied individual differences in thinking dispositions. For example, the Actively Openminded Thinking scale [8] measures the disposition to engage in rational thought, and the Rational-Experiential Inventory [9] assesses rational versus intuitive cognitive style. In addition to these self-report measures, there are several performance-based tests designed to measure analytic (vs. intuitive) cognitive style [10–13]. Analytic cognitive style is generally related to cognitive ability [14]. However, whereas measures of cognitive ability have been designed to capture people’s capacity to process complex information, measures of cognitive style were developed to assess one’s inclination to rely on analytic thinking to solve problems, particularly when an intuitive (but incorrect) answer is readily available. Expressed differently, measures of thinking dispositions are primarily designed to capture individual differences in goal management, epistemic values, and epistemic self-regulation [15]. It is therefore not surprising that measures of thinking dispositions can predict epistemically rational thinking above and beyond measures of cognitive ability [14,16].

Measures have recently been developed to try to isolate the “epistemic value component” of thinking dispositions [4, see also 3]. Values are generally conceived of as trans-situational goals that vary in importance and serve as guiding principles in people’s lives [17]. Thus, the epistemic value component of thinking dispositions should capture how important it is for people to adopt beliefs based on logic and the best available evidence. Building on social domain theory [18,19], and the domain theory of attitudes [20], which distinguish between the domains of personal preferences and moral concerns, Ståhl and colleagues further proposed that the value ascribed to epistemic rationality can be of a personal or moral nature [4]. That is, people can view it as more or less important to them personally that their beliefs are based on logic, and the best available evidence. However, they can also consider epistemic rationality as a universal moral good, and thereby believe that everyone *should* form their beliefs based on logic and evidence. The IRS and the MRS were developed to assess these two stances. Whereas the IRS assesses how important people think it is that their own beliefs are formed based on logic and evidence, the MRS measures the extent to which people view epistemic rationality as a moral issue. Scores on the IRS and MRS are moderately related, and stable over time [4]. Studies have also demonstrated that both of these measures are positively associated with belief in science, and negatively related to a number of epistemically suspect beliefs [4,6,7,21].

The observation that people can attach moral significance to epistemic rationality is important, as moral values and attitudes have unique properties. Unlike nonmoral values and attitudes, moral values and attitudes are generally perceived as objective facts, and considered to be universally applicable to everyone [22,23]. They are therefore uniquely strong predictors of attitude-consistent political action [24–27], as well as of social distancing from, and intolerance of, people with opposing views [28,29]. Consistent with such findings from the moral psychology literature, the MRS has been found to uniquely predict harsh moral judgments of, and social distancing from, people who make decisions based on an epistemically unreliable process (e.g., based on astrology, trust in alternative medicine, or religious faith). Moreover, the MRS uniquely predicts the willingness to financially support an organization that serves to prevent the spread of epistemically suspect beliefs [4]. Thus, just like other moral values and attitudes, moralized rationality motivates people to regulate their relationships, and to influence their communities more generally, in a value-consistent manner [30,31].

Whereas the MRS is a better predictor of intolerance of epistemic irrationality in others, the IRS appears to be a better predictor of cognitive performance. For example, the IRS (vs. MRS) is more reliably associated with indices of cognitive ability, and analytic cognitive style [6,7]. The IRS (vs. MRS) is also more strongly associated with belief in Science [4]. Moreover, the IRS moderates the association between cognitive ability and various epistemically suspect beliefs, such as paranormal and conspiracy beliefs [6]. Specifically, cognitive ability was more strongly negatively associated with paranormal beliefs and conspiracy beliefs among those who scored high (vs. low) on the IRS. Although correlational, these findings are consistent with the notion that attaching personal value to epistemic rationality provides people with the motivation needed to engage their cognitive faculties in pursuit of the truth. When people are committed to pursuing the truth, their cognitive ability should predict the epistemic quality of their conclusions. By contrast, when people are not motivated to pursue the truth, their cognitive ability should be less related to the quality of their conclusions, as those faculties are likely to either remain disengaged, or engaged in belief confirmation. Consistent with this interpretation, the negative association between cognitive ability and supernatural beliefs becomes stronger when the goal to be rational has been made more salient experimentally [32].

In summary, the available evidence suggests that ascribing personal value to epistemic rationality may motivate people to engage their cognitive faculties in pursuit of epistemically rational conclusions. By contrast, ascribing moral significance to epistemic rationality may motivate people to try to eradicate epistemically irrational beliefs and behaviors displayed by their fellow community members. Notably, however, very little is known about the psychological profiles of those who ascribe personal and/or moral value to epistemic rationality, and how they differ from those who do not. In this article we begin to address this question by examining how the MRS and IRS relate to the Big Five personality traits.

## Epistemic values and the Big Five

There are reasons to suspect that some Big Five personality traits may contribute to the personal and moral value ascribed to epistemic rationality. The most plausible candidate is arguably Openness to experience. Openness to experience breaks down into two aspects: Intellect and Openness. Intellect primarily captures curiosity about ideas, and thinking dispositions, and is also associated with fluid intelligence [33,34]. By contrast, Openness is thought to capture curiosity in other domains (e.g., aesthetics), and is associated with crystallized intelligence [33,34]. Because of its associations with interest in ideas, and thinking dispositions, it seems plausible that Openness to experience, and particularly Intellect, should predict how much value people ascribe to epistemic rationality. Based on previous studies indicating that it is primarily the IRS that is associated with an analytic cognitive style and cognitive ability [6,7], we further expect these relationships to be stronger for the IRS than for the MRS.

Could Big Five personality traits also help discriminate between those who ascribe personal versus moral value to epistemic rationality? There are theoretical, as well as indirect empirical reasons to think that Agreeableness may play such a role. Specifically, we propose that low Agreeableness may be uniquely associated with ascribing *moral* value to epistemic rationality. As outlined above, individuals who moralize rationality are harsh in their moral judgments of, and inclined to distance themselves from, individuals who hold or act upon epistemically irrational beliefs. Judging people harshly for their epistemically irrational beliefs and actions can arguably be considered insensitive and be viewed as a way of “punching down”. It therefore seems plausible that coming to moralize epistemic rationality may require a relatively disagreeable constitution. Notably, men score higher than women on the MRS on average [4]. In fact, being male is the only demographic that has consistently been associated with higher scores on the MRS in previous studies. This is noteworthy, as Agreeableness is also the Big Five trait on which men and women differ the most [35]. It seems plausible therefore that men’s (vs. women’s) relatively low levels of Agreeableness may contribute to their stronger inclination to moralize rationality. By contrast, people who score high (vs. low) on the IRS are not more inclined to judge people harshly for their epistemically irrational beliefs. This suggests that Agreeableness may be less related to the IRS than to the MRS, and thereby be a key difference in the psychological profile of people who ascribe moral versus personal value to epistemic rationality.

We examined the relationships between the Big Five traits on the one hand, and the IRS and MRS on the other, in two cross-sectional studies. The first study was conducted on Amazon’s Mechanical Turk. The second study was a replication, using the Psychology subject pool at a Midwestern public university. For brevity, and because procedures and materials were identical, we report the methods and results from both studies together.

## Method

### Participants

These studies were approved by the Office for the Protection of Research Subjects (OPRS) at the University of Illinois at Chicago (Protocol: 2018–1027).

#### Study 1

We requested 200 M-Turk workers residing in the U.S. Because some participants failed to click their way to the end-page of the survey, we ended up with a final sample of 222 participants (115 men, 105 women, 2 other, *M*_age_ = 35.69, *SD*_age_ = 10.51). The majority were Caucasian (72.5%), and the rest of the sample consisted of 10.8% African Americans 9% Hispanic/Latinos, 5.9% Asians, 0.5% Native Americans, and 1.4% other.

#### Study 2

Prior to the start of data collection, we decided to accept participants for this study until the end of the semester, which resulted in a sample of 345 participants from the Psychology department’s subject pool at the University of Illinois at Chicago (115 men, 230 Women, *M*_age_ = 19.27, *SD*_age_ = 1.71). The sample consisted of 35.1% Hispanic/Latinos, 28.7% Asians, 16.5% Caucasians, 9.3% African Americans, 0.3% Native Americans, and 10.1% other.

### Procedure and materials

Participants in Study 1 filled out the online survey at their own convenience, whereas participants in Study 2 were invited to come to the lab to take part in the study (seated in separate cubicles). Upon providing informed consent, and for those in the M-Turk sample, indicating that they were 18 years of age or older, participants were taken to an online survey that took approximately 30 minutes to complete. Participants received either $1.50 (Study 1), or course credit (Study 2) as compensation.

Except for one demographic question (level of education), materials were identical in both studies. Ascribed personal importance and moral value to epistemic rationality were assessed using the 6-item IRS and the 9-item MRS respectively [4]. The IRS contains items such as: “It is important to me personally to be skeptical about claims that are not backed up by evidence”, and “It is important to me personally to examine traditionally held beliefs using logic and evidence”. The MRS contains items such as: “Being skeptical about claims that are not backed up by evidence is a moral virtue”, and “Holding onto beliefs when there is substantial evidence against them is immoral”. All items were answered on a 7-point scale (1 = *Completely disagree*, 7 = *Completely agree*).

Big Five personality traits were assessed using the Big Five Aspects Scales [34]. This test consists of 20 items for each of the Big Five traits (Openness to experience, Conscientiousness, Extraversion, Agreeableness, Neuroticism). Each trait can be further broken down into two aspects: Openness to experience (Intellect, Openness), Conscientiousness (Industriousness, Orderliness), Extraversion (Enthusiasm, Assertiveness), Agreeableness (Compassion, Politeness), and Neuroticism (Volatility, Withdrawal). Each aspect is assessed using 10 items, and all responses are made on a 5-point scale (1 = *Very inaccurate*, 5 = *Very accurate*). After recoding reversed items, scores were averaged to create reliable measures of the Big Five traits, as well as of the ten aspects.

Finally, participants indicated their gender, age, race/ethnicity, and political orientation. In the M-Turk sample we also assessed level of education. After that, participants were thanked and compensated for their participation.

## Results

Means, standard deviations, and reliability statistics for all scales, as well as zero-order correlations between all variables are presented in Table 1 (Study 1) and Table 2 (Study 2). To examine the relationships between the IRS and MRS on the one hand, and their potential predictors, we carried out a series of hierarchical regression analyses. Control variables were entered in Step 1. In the M-Turk sample (Study 1) we controlled for gender, age, and level of education. In the student sample (Study 2), where all participants were recruited from the Psychology undergraduate subject pool, we only controlled for gender and age. In Step 2 we entered the Big Five scales. We also followed up with more fine-grained analyses in which we replaced the Big Five scales with the ten aspects scales in Step 2 (Step 1 remained the same).

**Table 1:** Means, Standard deviations, Chronbach’s Alphas, and zero-order correlations (Study 1).

Variable	M	SD	α	1	2	3	4	5	6	7
1 IRS	5.70	1.00	.88	-						
2 MRS	4.16	1.17	.83	.26***	-					
3 Openness	3.70	.58	.84	.42***	.04	-				
4 Conscientiousness	3.63	.64	.88	.24***	-.10	.43***	-			
5 Extraversion	3.26	.65	.87	.08	.01	.43***	.53***	-		
6 Agreeableness	3.74	.69	.89	.18**	-.25***	.45***	.56***	.21**	-	
7 Neuroticism	2.74	.80	.92	-.11	.16*	-.37***	-.47***	-.57***	-.35***	-

*** *p* < .001,

** *p* < .01,

* *p* < .05.

**Table 2:** Means, Standard deviations, Chronbach’s Alphas, and zero-order correlations (Study 2).

Variable	M	SD	α	1	2	3	4	5	6	7
1 IRS	5.61	.84	.75	-						
2 MRS	3.98	.76	.64	.36***	-					
3 Openness	3.45	.43	.72	.33***	.06	-				
4 Conscientiousness	3.37	.51	.81	.22***	.04	.33***	-			
5 Extraversion	3.36	.55	.85	.12*	-.03	.38***	.36***	-		
6 Agreeableness	3.84	.45	.78	.18**	-.16**	.18**	.04	.09	-	
7 Neuroticism	3.07	.66	.89	-.05	.04	-.16**	-.27***	-.29***	-.04	-

*** *p* < .001,

** *p* < .01,

* *p* < .05.

### Predictors of the IRS

#### Study 1

As can be seen in Table 3, the Big Five traits accounted for approximately 20% of the variance in scores on the IRS. As expected, scoring high (vs. low) on the IRS was associated with higher levels of Openness to experience. In addition, scoring high (vs. low) on the IRS was also associated with higher levels of Conscientiousness, but somewhat lower levels of Extraversion. Agreeableness and Neuroticism were both unrelated to scores on the IRS. As can be seen in Table 4, these results were primarily attributable to strong positive relationships between the IRS and the Intellect aspect of Openness to experience, as well as the Orderliness aspect of Conscientiousness. The negative link between the IRS and Extraversion was primarily attributable to the Enthusiasm aspect.

**Table 3:** Results of hierarchical regression analyses predicting scores on the IRS.

Study	Predictor	*b*	*SE*	Δ*R*^2^
1				
	Step 1			.036
	Gender	-.093	.135	
	Age	.014*	.006	
	Education	.10^†^	.055	
	Step 2			.204
	Openness to Exp	.448***	.074	
	Conscientiousness	.243**	.086	
	Extraversion	-.191*	.084	
	Agreeableness	-.079	.083	
	Neuroticism	.054	.081	
2				
	Step 1			.029
	Gender	.298**	.095	
	Age	-.005	.026	
	Step 2			.168
	Openness to Exp	.239***	.046	
	Conscientiousness	.132**	.045	
	Extraversion	-.035	.047	
	Agreeableness	.151***	.042	
	Neuroticism	.070	.045	

*** *p* < .001,

** *p* < .01,

* *p* < .05,

^†^*p* < .10.

**Table 4:** Results of hierarchical regression analyses predicting scores on the IRS.

Study	Predictor	*b*	*SE*	Δ*R*^2^
1				
	Step 1			.036
	Gender	-.093	.135	
	Age	.014*	.006	
	Education	.10^†^	.055	
	Step 2			.253
	Intellect	.467***	.094	
	Openness	.057	.082	
	Industriousness	-.157	.118	
	Orderliness	.286**	.084	
	Enthusiasm	-.184^†^	.096	
	Assertiveness	-.018	.102	
	Compassion	.040	.105	
	Politeness	.036	.099	
	Volatility	-.020	.098	
	Withdrawal	.030	.117	
2				
	Step 1			.029
	Gender	.298**	.095	
	Age	-.005	.026	
	Step 2			.228
	Intellect	.144**	.051	
	Openness	.144**	.045	
	Industriousness	-.121*	.057	
	Orderliness	.229***	.045	
	Enthusiasm	-.091^†^	.049	
	Assertiveness	.058	.056	
	Compassion	.171**	.049	
	Politeness	.025	.047	
	Volatility	-.057	.054	
	Withdrawal	-.005	.062	

*** *p* < .001,

** *p* < .01,

* *p* < .05,

^†^*p* < .10.

#### Study 2

As can be seen in Table 3, the Big Five traits once again accounted for a substantial amount of variance in scores on the IRS (16.8%). Moreover, we closely replicated the positive relationships between the IRS on the one hand, and Openness to experience and Conscientiousness on the other. However, the negative relationship between the IRS and Extraversion obtained in the M-Turk sample did not emerge in the student sample. Finally, unlike in the M-Turk sample, we also found positive relationships between the IRS and Agreeableness. As can be seen in Table 4, the more fine-grained analysis indicated that both intellect and openness contributed to the positive link between the IRS and Openness to experience in the student sample. By contrast, only orderliness contributed to the positive link between the IRS and Conscientiousness. In fact, the industriousness aspect of Conscientiousness was negatively associated with the IRS. Finally, compassion fully accounted for the positive association between the IRS and Agreeableness in this sample.

Taken together, the two studies consistently show that the Big Five traits account for a substantial amount of variance in scores on the IRS (16–20%), and that Openness to experience is the trait that is most strongly associated with high scores the IRS. Scoring high on the IRS is also associated with high levels of Conscientiousness. Both studies also indicate that the aspects of Intellect and Orderliness are of particular relevance, whereas relationships to the aspects of Openness and Industriousness were inconsistent across studies. No other Big Five traits showed consistent relationships to the IRS across the two studies. Lastly, neither gender nor age showed consistent relationships to the IRS across studies, and level of education (Study 1) was only marginally positively associated with the IRS.

### Predictors of the MRS

#### Study 1

As can be seen in Table 5, the Big Five dimensions accounted for a substantial amount variance in scores on the MRS (7.7%), although considerably less than on the IRS. As expected, scoring high on the MRS was associated with higher levels of Openness to experience. However, and unlike the IRS, scoring high on the MRS was almost equally closely associated with low levels of Agreeableness. Unexpectedly, scoring high on the MRS was also associated with higher levels of Neuroticism. No other Big Five traits were related to the MRS. As can be seen in Table 6, a more fine-grained analysis indicated that the relationships between the MRS, Openness to experience, and Agreeableness, were primarily attributable to a positive association between MRS and the Openness aspect of Openness to experience, and a negative association between the MRS and the Compassion aspect of Agreeableness. This analysis also indicated that the MRS was positively associated with the Withdrawal aspect of Neuroticism, but negatively associated with the Volatility aspect of Neuroticism.

**Table 5:** Results of hierarchical regression analyses predicting scores on the MRS.

Study	Predictor	*b*	*SE*	Δ*R*^2^
1				
	Step 1			.087
	Gender	.335*	.154	
	Age	-.026***	.007	
	Education	.045	.062	
	Step 2			.077
	Openness to Exp	.267**	.090	
	Conscientiousness	.034	.105	
	Extraversion	.073	.102	
	Agreeableness	-.258*	.102	
	Neuroticism	.218*	.099	
2				
	Step 1			.039
	Gender	.288**		
	Age	-.041^†^		
	Step 2			.037
	Openness to Exp	.086^†^	.045	
	Conscientiousness	.050	.044	
	Extraversion	-.051	.045	
	Agreeableness	-.101**	.041	
	Neuroticism	.065	.043	

*** *p* < .001,

** *p* < .01,

* *p* < .05,

^†^*p* < .10.

**Table 6:** Results of hierarchical regression analyses predicting scores on the MRS.

Study	Predictor	*b*	*SE*	Δ*R*^2^
1				
	Step 1			.087
	Gender	.335*	.154	
	Age	-.026***	.007	
	Education	.045	.062	
	Step 2			.138
	Intellect	-.001	.115	
	Openness	.267**	.100	
	Industriousness	-.186	.144	
	Orderliness	.183^†^	.102	
	Enthusiasm	.046	.117	
	Assertiveness	.190	.124	
	Compassion	-.218^†^	.127	
	Politeness	-.138	.121	
	Volatility	-.263	.119	
	Withdrawal	.350	.143	
2				
	Step 1			.039
	Gender	.288**	.086	
	Age	-.041^†^	.024	
	Step 2			.051
	Intellect	.091^†^	.052	
	Openness	.004	.046	
	Industriousness	.012	.058	
	Orderliness	.039	.045	
	Enthusiasm	-.041	.049	
	Assertiveness	.010	.056	
	Compassion	-.048	.050	
	Politeness	-.081^†^	.048	
	Volatility	-.050	.054	
	Withdrawal	.129*	.063	

*** *p* < .001,

** *p* < .01,

* *p* < .05,

^†^*p* < .10.

#### Study 2

As can be seen in Table 5, the Big Five scales once again accounted for some variance in scores on the MRS (3.7%), although yet again, considerably less than on the IRS. Replicating Study 1, the MRS was negatively associated with Agreeableness, and (marginally) positively related to Openness to experience. The unexpected association with Neuroticism obtained in Study 1 did not replicate. As shown in Table 6, analyses including the 10 aspects scales suggested that the MRS was primarily associated with the Intellect and Politeness aspects of Openness to experience and Agreeableness, although both of these associations were only marginally significant. As in Study 1, there was also a positive association between the MRS and the Withdrawal aspect of Neuroticism.

In conclusion, the Big Five traits accounted for variance in scores on both the IRS and the MRS in both studies. As expected, results from both studies suggest that people who score high on the IRS and MRS have in common that they tend to score relatively high on Openness to experience. Notably, the association with Openness to experience was considerably stronger for the IRS than for the MRS in both studies. Analyses of the two aspects of Openness to experience consistently showed that the IRS is associated with intellect, whereas the association with Openness varied between studies. For the MRS, analyses of the aspects of Openness to experience were inconsistent across studies.

Another consistent finding across studies is that scoring high on the MRS, but not on the IRS, is associated with low levels of Agreeableness. In fact, Agreeableness was the strongest predictor of scores on the MRS in Study 2, and comparable in strength to Openness to experience in Study 1. However, analyses at the aspects level did not produce consistent results across studies. Consistent with previous studies [4], men scored significantly higher on the MRS than women in both of the present studies (*d* = .32 to .37). Surprisingly, however, follow up analyses suggest that this gender difference cannot be attributed to differences in Agreeableness. When controlling for Agreeableness (and the other Big Five traits), the gender difference in scores on the MRS remains virtually identical in size in Study 1 (*b* = -.32, *p* < .05), as well as in Study 2 (*b* = -.28, *p* = .002).

Finally, scoring high on the IRS, but not on the MRS, was associated with higher levels of Conscientiousness in both studies. This association was in turn attributable to the Orderliness aspect of Conscientiousness.

## Discussion

People differ in how much personal and moral value they ascribe to epistemic rationality, and these individual differences are associated with distinct cognitive, affective, and behavioral outcomes. In the present research we begun to investigate where these epistemic values may stem from, as we examined how they relate to the Big Five personality traits. Evidence from two correlational studies consistently indicate that, out of all of the Big Five traits, Openness to experience is the strongest predictor of how much personal value people ascribe to epistemic rationality (IRS). Both studies further showed that the aspect of Intellect was positively associated with scores on the IRS, whereas a positive relationship to the Openness aspect was only obtained in Study 2. The fact that Intellect was a more reliable predictor of scores on the IRS than was Openness is consistent with previous work showing that Intellect uniquely captures differences in thinking dispositions, and curiosity about ideas.

Openness to experience was also a significant predictor of how much moral value people ascribe to epistemic rationality (MRS). Notably, however, this association was substantially weaker, and only emerged when controlling for the other Big Five traits (the zero-order correlation was nonsignificant in both studies). This pattern is consistent with previous studies showing that the MRS is not reliably associated with cognitive style, or cognitive ability, and less strongly associated with belief in science than is the IRS. The most reliable predictor of scores on the MRS was instead Agreeableness. People low (vs. high) on Agreeableness were more inclined to moralize epistemic rationality. By contrast, no consistent relationship was found between Agreeableness and the IRS across the two studies. This pattern of results was anticipated based on the observation that people who moralize rationality are uniquely willing to judge other people harshly for their epistemically suspect beliefs [4], as well as based on the fact that men (vs. women) generally score lower on Agreeableness [35], and higher on the MRS [4]. Notably, men did indeed score higher on the MRS than women in the present studies. However, this gender difference could not be accounted for by differences in Agreeableness, or any of the other Big Five traits. Future studies are needed to determine why men and women differ in their inclination to moralize epistemic rationality.

We also found consistent evidence across studies that Conscientiousness is positively associated with the IRS (but not the MRS), and that this association was attributable to the Orderliness aspect. Although this relationship was unanticipated, it seems reasonable in retrospect that people who strongly prefer order in their lives also strive to form beliefs based on logic and evidence. No other Big Five traits were consistently associated with scores on the IRS or MRS across studies.

### Limitations and implications

To the best of our knowledge, the present studies are the first to systematically examine how personal and moral epistemic values relate to basic personality traits. These studies thereby provide valuable initial insights regarding where epistemic values may stem from. We hasten to say, however, that these studies relied exclusively on cross-sectional correlational designs, and do not permit any conclusions about causality. Longitudinal studies, tracking the development of epistemic values over time, are needed to demonstrate that Openness to experience, low Agreeableness, and Conscientiousness indeed play a causal role in the emergence of personal and moral epistemic values. It is also important to note that the present studies were restricted to American M-Turk workers and university students. Future studies are therefore needed to determine to what extent the present findings generalize to other populations and cultures.

Although the Big Five personality traits were a reasonable place to start the search for antecedents of these epistemic values, there are other plausible candidates to be examined as well. For example, we suspect that the Honesty/humility dimension from the HEXACO model of personality [36] may play a role in the development of these values. Being intellectually honest about the quality of evidence in support of (vs. in opposition to) one’s beliefs is at the heart of being epistemically rational. It seems reasonable to assume that people who live by the values of honesty and humility more generally, should be more inclined to endorse these values in the context of belief formation as well.

Although the present findings suggest that low levels of Agreeableness, and high levels of Openness to experience may be important antecedents to the moralization of epistemic rationality, it is not clear what the psychological process is. Based on the existing literature on moralization, we know that information and imagery that evokes strong moral emotions play a key role in the moralization of previously nonmoral stances [37–40]. Furthermore, there is some evidence that moral emotions can directly lead to moralization of previously nonmoral stances, as well as through a cognitive process of moral piggybacking [41], whereby moral emotions can cause a previously nonmoral stance to become integrated with broader, pre-existing moral values or principles, and thereby take on new moral significance [38]. Future research is needed to determine whether similar affect-driven processes cause moralization of epistemic rationality as well, and if so, with what broader moral values or principles epistemic rationality may become integrated. Values such as honesty (cf. intellectual honesty), and Sanctity/degradation (cf. intellectual purity) strike us as plausible candidates worthy of investigation.

## Supporting information

S1 Text List of variables in each dataset. (DOCX)

S1 File Study 1 dataset. (SAV)

S2 File Study 2 dataset. (SAV)
