# Supplementary material for: Epistemic values and the Big Five: Personality characteristics of those who ascribe personal and moral value to epistemic rationality
Source: PLoS One. 2021 Oct 5;16(10):e0258228. doi: 10.1371/journal.pone.0258228 (PMC8491882; doi:10.1371/journal.pone.0258228)
Supplement: S1 Text — (DOCX) [file pone.0258228.s001.docx]

**Study 1: Variable List**

MR 1-9 = Moralized Rationality Scale items (MRS)

IR 1-6 = Importance of Rationality Scale items (IRS)

Vol 1-10 = Volatility items (BFAS)

Wit 1-10 = Withdrawal items (BFAS)

Com 1-10 = Compassion items (BFAS)

Pol 1-10 = Politeness items (BFAS)

Ind 1-10 = Industriousness items (BFAS)

Ord 1-10 = Orderliness items (BFAS)

Ent 1-10 = Enthusiasm items (BFAS)

Ass 1-10 = Assertiveness items (BFAS)

Int 1-10 = Intellect items (BFAS)

Ope 1-10 = Openness items (BFAS)

Gender = Gender

Age = Age

Race = Race/ethnicity

Education = Level of education

RelID = Religious identity

JewishID = Jewish denomination

ChristID = Christian denomination

OtherRel = Other religious identity

NonRel = Nonreligious identity

PoSoc = Political orientation on social issues

PoEco = Political orientation on economic issues

MR 8-9rec = Recoded (previously reversed MRS items)

MRS = Moralized Rationality Scale (average)

IRS = Importance of Rationailty Scale (average)

Volrec = Recoded (previously reversed) Volatility items

Witrec = Recoded (previously reversed) Withdrawal items

Comrec = Recoded (previously reversed) Compassion items

Polrec = Recoded (previously reversed) Politeness items

Indrec = Recoded (previously reversed) Industriousness items

Ordrec = Recoded (previously reversed) Orderliness items

Entrec = Recoded (previously reversed) Enthusiasm items

Assrec = Recoded (previously reversed) Assertiveness items

Intrec = Recoded (previously reversed) Intellect items

Operec = Recoded (previously reversed) Openness items

Volatility = Volatility scale (average)

Withdrawal = Withdrawal scale (average)

Compassion = Compassion scale (average)

Politeness = Politeness scale (average)

Industriousness = Industriousness scale (average)

Orderliness = Orderliness scale (average)

Enthusiasm = Enthusiasm scale (average)

Assertiveness = Assertiveness scale (average)

Intellect = Intellect scale (average)

Openness = Openness scale (average)

ZMRS = Standardized MRS scale

ZIRS = Standardized IRS scale

ZVolatility = Standardized Volatility scale

ZWithdrawal = Standardized Withdrawal scale

ZCompassion = Standardized Compassion scale

ZPoliteness = Standardized Politeness scale

ZIndustriousness = Standardized Industriousness scale

ZOrderliness = Standardized Orderliness scale

ZEnthusiasm = Standardized Enthusiam scale

ZAssertiveness = Standardized Assertiveness scale

ZIntellect = Standardized Intellect scale

ZOpenness = Standardized Openness scale

Neuroticism = Neuroticism scale

Agreeableness = Agreeableness scale

Conscientiousness = Conscientiousness scale

Extraversion = Extraversion scale

OpennesstoExp = Openness to experience scale

ZNeurotisism = Standardized Neuroticism scale

ZAgreeableness = Standardized Agreeableness scale

ZConscientiousness = Standardized Conscientiousness scale

ZExtraversion = Standardized Extraversion scale

ZOpennesstoExp = Standardized Openness to experience scale

GenderDummy = Gender dummy-coded

**Study 2: Variable List**

MR 1-9 = Moralized Rationality Scale items (MRS)

IR 1-6 = Importance of Rationality Scale items (IRS)

Vol 1-10 = Volatility items (BFAS)

Wit 1-10 = Withdrawal items (BFAS)

Com 1-10 = Compassion items (BFAS)

Pol 1-10 = Politeness items (BFAS)

Ind 1-10 = Industriousness items (BFAS)

Ord 1-10 = Orderliness items (BFAS)

Ent 1-10 = Enthusiasm items (BFAS)

Ass 1-10 = Assertiveness items (BFAS)

Int 1-10 = Intellect items (BFAS)

Ope 1-10 = Openness items (BFAS)

Gender = Gender

Age = Age

Race = Race/ethnicity

Education = Level of education

RelID = Religious identity

JewishID = Jewish denomination

ChristID = Christian denomination

OtherRel = Other religious identity

NonRel = Nonreligious identity

PoSoc = Political orientation on social issues

PoEco = Political orientation on economic issues

MR 8-9rec = Recoded (previously reversed MRS items)

Volrec = Recoded (previously reversed) Volatility items

Witrec = Recoded (previously reversed) Withdrawal items

Comrec = Recoded (previously reversed) Compassion items

Polrec = Recoded (previously reversed) Politeness items

Indrec = Recoded (previously reversed) Industriousness items

Ordrec = Recoded (previously reversed) Orderliness items

Entrec = Recoded (previously reversed) Enthusiasm items

Assrec = Recoded (previously reversed) Assertiveness items

Intrec = Recoded (previously reversed) Intellect items

Operec = Recoded (previously reversed) Openness items

MRS = Moralized Rationality Scale (average)

IRS = Importance of Rationailty Scale (average)

Volatility = Volatility scale (average)

Withdrawal = Withdrawal scale (average)

Compassion = Compassion scale (average)

Politeness = Politeness scale (average)

Industriousness = Industriousness scale (average)

Orderliness = Orderliness scale (average)

Enthusiasm = Enthusiasm scale (average)

Assertiveness = Assertiveness scale (average)

Intellect = Intellect scale (average)

Openness = Openness scale (average)

Neuroticism = Neuroticism scale

Agreeableness = Agreeableness scale

Conscientiousness = Conscientiousness scale

Extraversion = Extraversion scale

OpennesstoExp = Openness to experience scale

ZNeurotisism = Standardized Neuroticism scale

ZAgreeableness = Standardized Agreeableness scale

ZConscientiousness = Standardized Conscientiousness scale

ZExtraversion = Standardized Extraversion scale

ZOpennesstoExp = Standardized Openness to experience scale

GenderDummy = Gender dummy-coded

ZVolatility = Standardized Volatility scale

ZWithdrawal = Standardized Withdrawal scale

ZCompassion = Standardized Compassion scale

ZPoliteness = Standardized Politeness scale

ZIndustriousness = Standardized Industriousness scale

ZOrderliness = Standardized Orderliness scale

ZEnthusiasm = Standardized Enthusiam scale

ZAssertiveness = Standardized Assertiveness scale

ZIntellect = Standardized Intellect scale

ZOpenness = Standardized Openness scale

ZMRS = Standardized MRS scale

ZIRS = Standardized IRS scale
